# Propensity Score-Matched Analysis of Single Fraction Robotic Radiosurgery Versus Open Partial Nephrectomy in Renal Cell Carcinoma: Oncological Outcomes

**DOI:** 10.7759/cureus.21623

**Published:** 2022-01-26

**Authors:** Michael Staehler, Tina Schuler, Annabel Spek, Severin Rodler, Alexander Tamalunas, Christoph Fürweger, Alexander Muacevic

**Affiliations:** 1 Urology, University Hospital, Ludwig Maximilians University Munich, Munich, DEU; 2 Medical Physics, European CyberKnife Center, Munich, DEU; 3 Stereotaxy and Neurosurgery, University Hospital Cologne, Cologne, DEU; 4 Radiosurgery, European CyberKnife Center, Munich, DEU

**Keywords:** stereotactic ablative radiation therapy, sbrt, radiation therapy, ablation therapy, rrs, sabrt, open partial nephrectomy, renal cell carcinoma

## Abstract

Introduction

High-dose local stereotactic robotic radiosurgery (RRS) is a non-invasive alternative to surgery in renal masses and selected patients. We have, so far, limited its use to the elderly and patients at high risk from surgery. In this study, we matched patients with renal tumors who were treated with single fraction RRS to patients who underwent open partial nephrectomy (OPN).

Methods

Between January 2009 and December 2017, we included 571 consecutive patients undergoing OPN and 99 patients who underwent RRS in this retrospective analysis. Patients had to have a follow-up of at least six months and we were able to match 35 with a propensity score. Matching criteria were Eastern Cooperative Oncology Group (ECOG) status, age, clinical tumor, nodes, and metastases (TNM), and tumor diameter. Tumor response, renal function, survival, and adverse events were evaluated every three months until progression or death.

Results

Median age was 65 years for RRS (range 58-75) and 71 (range 56-76) for OPN (p=0.131). Median diameter of renal tumors was 2.8 cm (range 2.4-3.9) for RRS and 3.5 cm (2.8-4.5) for OPN, p=0.104. Median follow-up was 28.1 months (range 6.0-78.3 months). Local tumor control nine months after RRS and OPN was 98% (95% CI: 89-99%). Renal function remained stable with a median creatinine clearance (Chronic Kidney Disease Epidemiology Collaboration (CKD-EPI)) at baseline of 76.8mlmin/1.73m^2^ (range 25.3-126.3) and 70.3ml/min/1.73m^2^ (range 18.6-127.3) at follow-up (p=0.89). Median overall survival was not reached. No difference in overall survival (OS) was seen in RRS compared to OPN (p=0.459).

Conclusions

Single fraction RRS is an alternative to OPN in patients unfit for surgery. Oncological and functional results are comparable to those of OPN. Further studies are needed to determine long-term results and limits of RRS in this setting and in younger patients.

## Introduction

Renal Cell Carcinoma (RCC) is the deadliest urologic malignancy with 30-40% of patients dying of their cancer. Globally, the International Agency on Cancer Research estimates 144,000 deaths from kidney cancer in 2012 [1,2]. RCC accounts for 90% of all kidney cancers with upper tract urothelial cancer being the second most common entity of renal neoplasm [1]. Of the patients, 65% are diagnosed with localized disease that is confined to the kidney and 35% are diagnosed with the disease spread beyond the kidney, with 16% showing spread to distant organs [1].

Patients diagnosed with localized disease who receive surgery and have a low-risk disease have a five-year disease-specific survival rate of 97%. The incidence of kidney cancer has increased over the last three decades, with increased detection of early-stage disease [1,2].

Surgical removal with either partial nephrectomy (PN) or radical nephrectomy (RN) remains the standard of care in localized RCC [3-5]; however, increasing incidence and subsequent need for surgical intervention have given rise to less invasive alternatives, including local ablative procedures like radiofrequency ablation (RFA) or cryotherapy. Active surveillance is also an option for elderly patients as well as young patients who are ineligible for surgery, harboring small renal masses (SRM) <4cm [6-9].

High-dose local stereotactic robotic radiosurgery (RRS) has been recently introduced as a non-invasive alternative to surgery in renal masses. We compared patients with renal tumors who were treated with single fraction RRS to patients who underwent open partial nephrectomy (OPN). To eliminate selection bias and compare oncological efficacy, we used propensity score matching (PSM) to analyze these different treatment approaches.

This work was presented in part as a poster at the Annual Meeting of the American Association of Urology 2019 held on May 3-6, 2019, in Chicago, United States.

## Materials and methods

Study design 

Data were identified retrospectively by scrutinizing our prospectively maintained database after acquiring the approval of the institutional review board and ethics committee. Out of 571 consecutive patients undergoing OPN and 99 patients who underwent RRS, we could match 35 with a propensity score between January 2009 and December 2017. The indication for RRS was determined by the oncological tumor board with the patients’ oncological status and their treatment preference being the most important factors. All patients had to have histological proof of clear cell RCC. Matching criteria were Eastern Cooperative Oncology Group (ECOG) status, age, clinical tumor, nodes, and metastases (TNM), and tumor diameter. Tumor response, renal function, survival, and adverse events were estimated every three months with a follow-up of at least six months.

Statistical analysis 

The PSM method was applied to eliminate any significant difference in oncological and clinical characteristics. Nonparsimonious and multivariate logistic regression was utilized to calculate the propensity scores on the basis of ECOG performance status, age, and tumor size. In 64 patients undergoing RRS, no suitable pair could be identified; thus 35 patients in the RRS group were perfectly matched to 35 patients in the OPN group in a 1:1 ratio according to the nearest neighbor matching method.

Calculations were performed using the IBM SPSS Statistics for Windows, Version 26.0 (Released 2019. IBM Corp., Armonk, New York). Kaplan-Meier analysis, Cox regression, and log-rank calculations were used to estimate progression-free survival (PFS) and overall survival (OS) with a one-sided p-value. Differences in matching categories were calculated with the Mann-Whitney U test and statistical significance was set at a two-sided p<.05.

Radiosurgery and open partial nephrectomy

RRS was performed using a radiosurgical device (Cyberknife® G6, Accuray Incorporated, Sunnyvale, California, United States) with real-time tumor tracking [10]. Integral to the system are orthogonally positioned x-ray cameras, which acquire images during treatment. The images are processed automatically to identify radiographic features and registered to the treatment planning study to measure the real-time position of the treatment site. The system adapts to changes in patient position during treatment by acquiring targeting images repeatedly and then adjusting the direction of the treatment beam. In contrast to a gantry-mounted linear accelerator (LINAC), the treatment beam can be directed at the target from nearly anywhere around the patient, limited only by obstacles such as the treatment couch. The robot is controlled such that the radiation beam always points to the tumor [11,12]

Prior to radiation, three gold fiducials (CP Medical Inc., Norcross, Georgia, United States) were placed next to the renal tumor via an 18G needle under local anesthesia and ultrasound guidance in the Urology outpatient center. Abdominal CT (arterial and venous phase) and gadolinium-enhanced MRI were used to identify the three-dimensional (3D) target volume. Additionally, a safety margin (4 mm) was added to the tumor diameter in all three dimensions. In a single-session treatment, a total of 25 Gy to the 70% isodose (dose maximum 34-37 Gy) were administered to the target volume, using a 6MV compact LINAC mounted on a six-axis robotic manipulator. All patients were pretreated with ondansetron on the day of treatment to prevent nausea due to radiation. During treatment, respiratory motion tracking of the target volume was done as previously described [11,12]. The whole procedure lasted about 40 minutes, and patients were discharged from the institute immediately after the treatment.

OPN was performed according to the surgeon’s discretion following standard surgical principles. 

## Results

**Figure 1:**
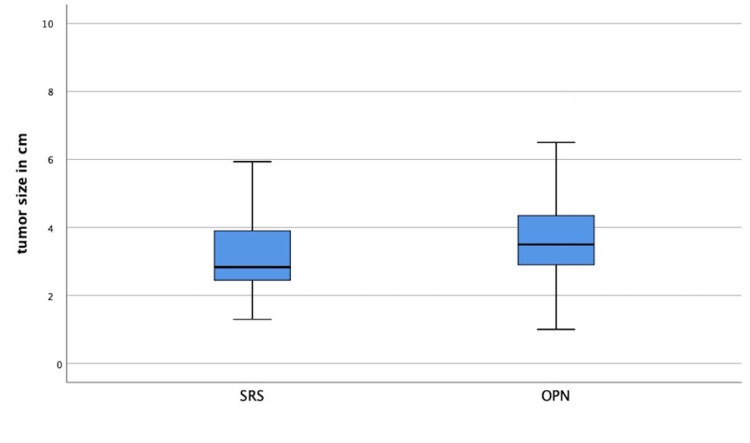
Tumor size in the groups (p=0.104)

Thirty-five patients were identified to be included in the PSM analysis. The difference in matching criteria ECOG, stage, age, and tumor size were not statistically significant between the groups with p-values of 0.232, 0.788, 0.131, and 0.104, respectively. For example, Figure 1 shows the tumor size in both groups.

Median age was 65 years for RRS (range 58-75) and 71 (range 56-76) for OPN (p=0.131). Median diameter of renal tumors was 2.8 cm (range 2.4-3.9) for RRS and 3.5 cm (2.8-4.5) for OPN, p=0.104. Median follow-up was 28.1 months in both groups (range 6.0-78.3 months). Tumor grading and R.E.N.A.L- Nephrometry Score were not predictive of outcome. RRS led to a complete response in nine patients, a partial response in 24, and stable disease in seven patients. Local tumor control nine months after RRS and OPN was 98% respectively (95% CI: 89-99%). One patient experienced local recurrence seven years after RRS, treated by repeat RRS. 

**Figure 2:**
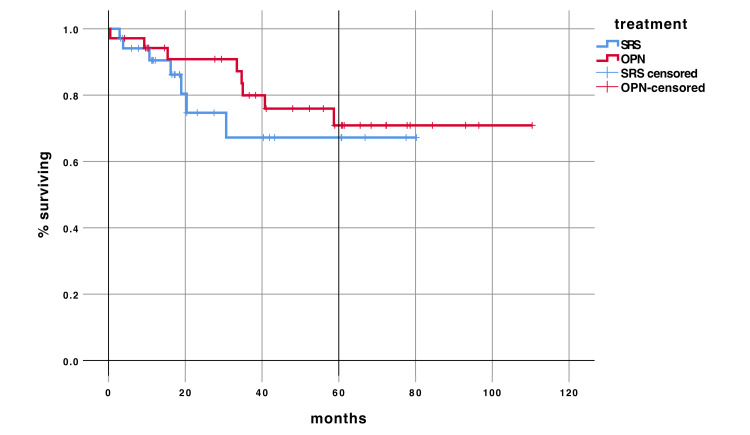
Overall survival compared in OPN and RRS, p = 0.461 OPN: Open Partial Nephrectomy; RSS: Robotic Radiosurgery; SRS: Stereotactic Radiosurgery

Median OS was not reached so far. No difference in OS was seen in the RRS compared to OPN with a hazard ratio of 1.48 (95% CI 0.5-4.16) (p=0.461) (Figure 2). 

Renal function remained stable with a median creatinine clearance (Chronic Kidney Disease Epidemiology Collaboration (CKD-EPI)) at baseline of 76.8mlmin/1.73m^2^ (range 25.3-126.3) and 70.3ml/min/1.73m^2^ (range 18.6-127.3) at follow-up (p=0.89). No major complications (Clavien-Dindo > grade II) were recorded in either group. 

## Discussion

Guidelines for the treatment of RCC recommend PN as the standard of care for the treatment of localized renal masses suspicious for RCC [11]. However, many patients with SRMs are poor surgical candidates due to age, compromised renal function, and other comorbidities. Active surveillance, RFA, and cryoablation are some of the alternative options recommended for these patients [1]. We present data on SRM in an elderly patient population who were not good surgical candidates. Thus, it might be questioned whether they had an indication for intervention or not. The recurrence and metastatic potential of SRM are higher than historically thought and has to be estimated at about 10%, urging for intervention if the expected life span of a patient is longer than five years [1,12,13]. 

The question remains which ablation technique is superior in terms of oncological outcome, safety, and limitations of usability. Data in available literature suggest that RFA might be inferior to cryoablation and the largest series comparing it to PN showed inferior oncological outcomes [1,14,15]. A systemic review on SRM therapy using cryoablation revealed that cryoablation has a lower cancer-specific and overall survival than PN, although follow-up is limited to less than three years in most of the series (20-27.8 months). Complications are seen in 7.3-29.8% with up to 10.9% being major [16]. In a matched-pair analysis looking at cryoablation versus PN, a significantly higher local recurrence rate was seen in T1b tumors [17]. Cancer-specific survival is also shorter with cryotherapy compared to PN in pT1b tumors. In pT1a this difference was not clinically relevant [18].

It is well known that tumor size is not predictive of oncological outcomes in SRM [19,20]. Even as our matched cohorts tend to show a slightly larger median tumor size in OPN without statistically significant difference, the likelihood of oncological aggressiveness cannot be assessed based on diameter alone. 

RFA and cryoablation have limitations when it comes to the localization of tumors. The proximity of the renal tumor to major vessels, the collecting system, and the bowels are seen as a contraindication for these procedures as well as the central location in the kidney [18]. RRS has no limitations if it comes to location. Previous studies also show that it is feasible to treat upper tract urothelial cancer within the kidney without causing urinary fistulas, which is not possible with RFA or cryotherapy [21-23]. Functional outcomes for RRS so far have been excellent and reported prior to this analysis [22]. There was no functional impairment in our series and long-term functional outcome is excellent even in solitary renal units as reported previously [21].

Recently published data revealed that distress in patients with RCC is independent of the stage or size of the tumor [24]. Stereotactic radiosurgery (SRS) could be an excellent alternative to active surveillance for patients who might experience significant emotional distress and would prefer intervention over routine scanning.

**Figure 3:**
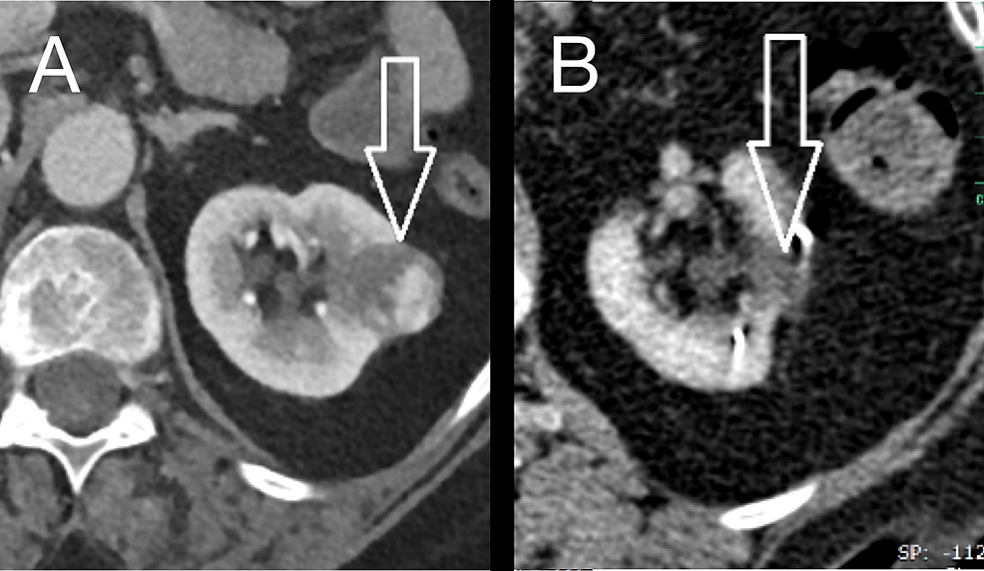
Complete response of RCC treated by RRS: (A) baseline and (B) at one-year follow-up RCC: Renal Cell Carcinoma; RRS: Robotic Radiosurgery

**Figure 4:**
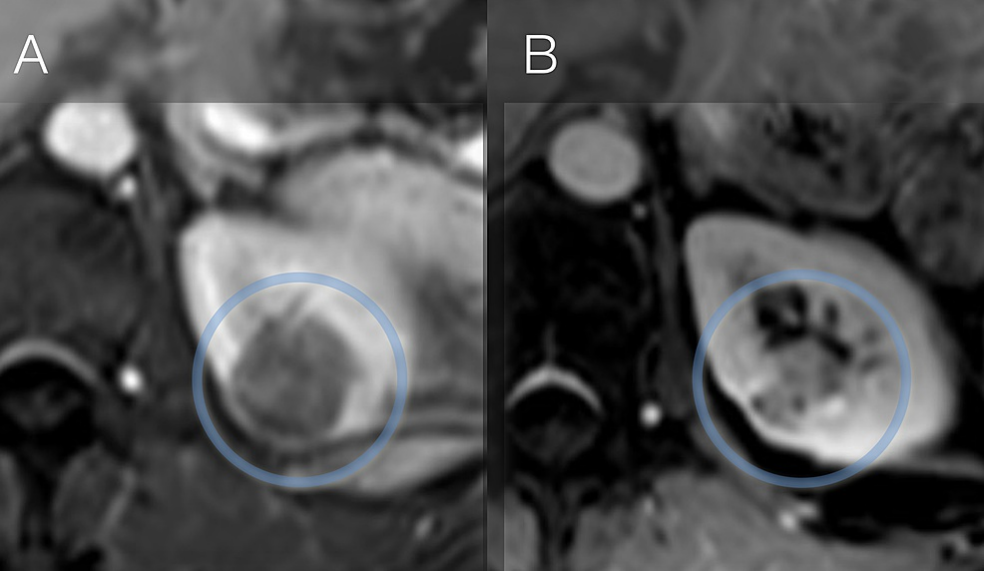
Result of RRS therapy in a patient with 3.2cm left-sided RCC, RENAL Score 9: (A) baseline and (B) at one-year follow-up. Although tumor remains detectable on imaging, typical cystic transformation can be seen as a reaction to the high dose radiation RRS: Robotic Radiosurgery; RCC: Renal Cell Carcinoma

Ablation techniques including RRS do not offer the same degree of tissue removal as surgical resection [1]. Estimation of response might be complicated as the majority of lesions will remain visible on subsequent scans. As shown in Figure 3, tumors might completely vanish; however, it remains unclear how long that takes. Some tumors (Figure 4) might still be visible but typically change towards a more cystic appearance showing micro-cysts and perfusion alterations as a consequence of radiation exposure. Little is known about which tumors might completely disappear, and which ones might not. Additional analysis is needed; however, patient reluctance to undergo additional biopsy after RRS therapy is an impediment to better understanding. 

Radiological evaluation is further complicated by the fact that perfusion changes are not as quantifiable as they are after cryotherapy or RFA. Therefore, we recommended response evaluation criteria in solid tumors (RECIST) criteria to categorize changes in the tumor’s size, which will lead to the classification of partial response (diameter reduced by at least 30%) in the majority of lesions treated by RRS [22,25].

In this setting, local recurrence has to be defined as a new onset of growth of the renal tumor treated. Of the patients in our dataset, only one local recurrence was identified. The uncertainty of size urges for survival data, which we could show to be no different compared to PN in our dataset.

One important consideration is the dosing of the RRS therapy. Based on a recently published multi-center analysis, we revealed that the lower the number of fractions and higher the doses the better the tumor control [25,26]. Thus, we recommend and used 25Gy to the 70 percentile in one fraction for all of the patients included in this analysis. The RRS system we used is particularly suited for a single-session approach as the unique tracking capabilities enable real-time tumor tracing in moving organs with an accuracy below 1 mm. 

Previous data have shown, that complications with RRS are extremely rare and we have not seen any major complications in this series of patients. The main complication is fatigue on the day following the procedure.

Literature shows data comparing PN with percutaneous or laparoscopic cryoablation with mixed oncological outcomes. Despite two trials showing no difference in OS, cancer-specific survival, local recurrence, or progression to metastatic disease, three trials revealed significant benefit for surgical approach in either one of these outcomes [27].

Our data are the first to show oncological equivalence in a PSM cohort of patients treated with single fraction RRS. Thus, RRS harbors the potential of an alternative to surgery in selected patients without the limitations of thermal ablation techniques. 

Limitations in the literature so far are the lack of prospective data and the selection bias of patients. Especially as ablative techniques are used in patients unfit for surgery, OS might not be the best parameter to measure the efficacy of any ablative therapy. Our dataset also suffers from that bias; however, in the end, it is not important whether patients get the best treatment but whether the treatment they get is best for them. Thus, data proving the equivalence of therapies are helpful. We also want to point out that there is a size limitation to single fraction RRS of below 5cm and that larger tumors are not suitable for this kind of therapy. Nonetheless, our dataset supports a broader indication and development of future trials.

## Conclusions

Single fraction RRS as an outpatient procedure is an alternative to OPN in selected patients. Oncological results are similar to those of OPN. Functional results are so far good. In patients at high risk for surgical complications, RRS seems to eliminate that risk. Further studies are needed to determine long-term results and limits of RRS in this setting and also in younger patients. As the data are superior to most ablation techniques, RRS should be considered in elderly and frail patients as a standard of care. 
